# Early vs. late treatment initiation in multiple sclerosis and its impact on cost of illness: A register-based prospective cohort study in Sweden

**DOI:** 10.1177/20552173221092411

**Published:** 2022-04-24

**Authors:** Korinna Karampampa, Hanna Gyllensten, Chantelle Murley, Kristina Alexanderson, Andrius Kavaliunas, Tomas Olsson, Ali Manouchehrinia, Jan Hillert, Emilie Friberg

**Affiliations:** Department of Clinical Neuroscience, 27106 Karolinska Institutet, Stockholm, Sweden; Department of Clinical Neuroscience, 27106 Karolinska Institutet, Stockholm, Sweden; Institute of Health and Care Science, 3570 University of Gothenburg, Gothenburg, Sweden; Department of Clinical Neuroscience, 27106 Karolinska Institutet, Stockholm, Sweden

**Keywords:** Multiple sclerosis, cost of illness, COI, early treatment, costs

## Abstract

**Background:**

Early treatment with disease modifying therapies (DMTs) for multiple sclerosis (MS) has been associated with lower disability progression; the aim was to explore its association with cost of illness (COI) in MS.

**Methods:**

All people with relapsing-remitting MS in the Swedish MS register, aged 20–57 years and receiving their first MS DMT in 2006–2009, were followed in nationwide registers for 8 years. Healthcare costs (in- and outpatient healthcare, DMTs and other prescribed drugs), and productivity losses (sickness absence and disability pension) of individuals receiving therapy in ≤6 months after diagnosis (early treatment group) were compared to those receiving therapy >6 months (late treatment group). Using Poisson regressions, the mean COI per patient per year, and per group, was estimated, adjusted for disability progression.

**Results:**

The early treatment group comprised 74% of the 1562 individuals included in the study. The early treatment group had lower productivity losses over time. Both groups had similar healthcare costs, which first increased and then decreased over time.

**Conclusions:**

Early DMT in MS could result in lower productivity losses possibly through maintained work capacity. COI serves as an objective measure showing the advantage of early vs. late treatment initiation in MS.

## Introduction

Multiple sclerosis (MS) is an often progressive, neurological condition,^1^ classified into different forms; relapsing-remitting MS, secondary-progressive MS, or primary-progressive MS.^2^ Sweden has the 2^nd^ highest prevalence of MS in Europe at 189 cases per 100,000.^3^

Since MS is diagnosed in early adulthood, usually when aged 20–40 years,^1^ it can affect people's work capacity.^4^ About 43% of people with MS (PwMS) who not were in paid work had quit their employment within the first three years after diagnosis.^4^ In fact, productivity losses account for 65%-75% of all costs in MS,^5,6^ due to elevated rates of sickness absence (SA) and/or disability pension (DP).^7–9^

Costs for disease modifying therapies (DMTs), inpatient, and specialized outpatient care are also substantial among PwMS.^7^ The annual cost of illness (COI) of MS has been shown to increase with disability progression.^7^ DMTs aim to slow disease progression, and therefore, can potentially slow the progression of the COI.^10^

However, the timing of DMT initiation is of essence; early MS therapy can slow the accumulation of disability early-on, leading to better clinical outcomes over time.^10,11^ It also improves capacity to maintain work^12^ and is associated with better socioeconomic outcomes.^13^ Therefore, early therapy could also have a positive impact on the overall COI in MS. However, no such longitudinal studies have been conducted.

The aim of this study was to explore the association between the timing of DMT initiation in relation to MS diagnosis, i.e., early vs. late therapy, with the overall COI of MS in Sweden.

## Material and methods

This was a register-based, longitudinal cohort study. Microdata, linked using the unique personal identity number that all residents in Sweden are assigned, was obtained from the following Swedish nationwide registers, kept by four authorities:

- Region Stockholm:
  - Swedish MS register (SMSreg)^14^: Was used to identify the cohort members and for information on MS diagnosis date, type of MS, information on MS disability (Expanded Disability Status Scale, EDSS), and type and date of DMTs.
- National Board of Health and Welfare:
  - National Patient Register (NPR)^15^: Dates and diagnoses for inpatient and specialized outpatient healthcare.
  - Swedish Prescribed Drug Register (SPDR)^15^: Dates, names, and costs for all prescribed drugs dispensed at pharmacies.
  - Cause of Death Register (CDR)^15^: Year of death.
- Statistics Sweden: Longitudinal Integration Database for Health Insurance and Labor Market Studies (LISA)^15^: Sex, birth year, educational level, country of birth, type of living area, and family situation.
- Swedish Social Insurance Agency^15^: Micro Data for the Analysis of Social Insurance register (MiDAS): dates, diagnoses, and grade of SA and DP.

PwMS who had the relapsing-remitting form of MS (RRMS), initiated their first DMT treatment (interferons, glatiramer acetate, and natalizumab) in 2006–2009 (index year) after MS diagnosis was established, and when aged 20–57 years were identified from the SMSreg. All included were followed for 8 years in total; last year of follow-up was 2013–2016 (depending on their index year).

Never-users of DMTs, those without RRMS, individuals receiving DMT before being diagnosed with MS, and those who did not live in Sweden during the index year, were not included in the study.

Early (≤6 months) and late (>6 months) DMT groups were then defined in relation to the date of MS diagnosis. The cut-off value of 6 months was chosen arbitrarily based on information from previously published studies.^11,12^

The project was approved by the Regional Ethical Review Board of Stockholm, Sweden.

### Study outcomes

#### MS disability, sociodemographic characteristics, and multi-morbidity

MS disability was defined using EDSS scores.^16^ Scores range from 0 to 10, with 0.5 step intervals (0 indicating no impairment, while 10 indicating death from MS).^17^ A clinically meaningful change in the EDSS score is a change of at least one point in patients with EDSS <5.5 and 0.5 point for those with EDSS of ≥5.5.^18^

EDSS information is recorded in the SMSreg during visits to neurologists.^14^ According, when multiple EDSS scores within a calendar year were available, the highest EDSS value was retained. If there was no EDSS information recorded in a calendar year, the average EDSS score was used for that year, computed from the scores of PwMS in the same treatment group and index year.

The following sociodemographic characteristics were measured at the year when therapy started (index year): Sex, age, educational level, country of birth, type of living area, and family situation. Multi-morbidity in the index year was derived utilizing the Rx-Risk Comorbidity Index^19^ (cancer morbidity was not included), based on the type of prescribed drugs the PwMS bought, according to SPDR Using information from the Rx-Risk Comorbidity Index^19^ the existence of multi-morbidity (yes/no) was established as well as whether PwMS had been diagnosed with anxiety/depression (based on prescribed drugs; yes/no).

#### Healthcare costs and productivity losses

The average COI per patient per year was defined from a societal perspective, quantifying the healthcare resources consumed by PwMS, as well as their SA and DP, and then multiplying them with unit costs (Table 1). All healthcare resource consumption, SA, and DP during a calendar year, were included, irrespective of whether MS was listed as the main diagnosis.

**Table 1.** Unit costs used in the calculation of healthcare costs and productivity losses.

	Year	Value in 2018 SEK	Value in 2018 Euros^a^	Source
Average inpatient and outpatient cost per 1.0 DRG	2006	50,973 kr	4969 €	Swedish Association of Local Authorities and Regions [Sveriges Kommuner och Landsting], KPP Somatik vård^20^
	2007	50,224 kr	4896 €	
	2008	51,388 kr	5009 €	
	2009	51,785 kr	5048 €	
	2010	50,457 kr	4919 €	
	2011	50,286 kr	4902 €	
	2012	49,820 kr	4857 €	
	2013	51,468 kr	5017 €	
	2014	53,388 kr	5204 €	
	2015	55,807 kr	5440 €	
	2016	57,334 kr	5589 €	
Co-payment for hospital stay (cost per day of stay)	2018	100 kr	10 €	Assume 100 SEK per day, as this is the case for the majority of the regions in Sweden (including Stockholm).^21^ The max co-payment amount for inpatient care was set to 1500 SEK per year (assumption for whole Sweden, based on information from the region Västra Götaland)^22^
Co-payment for visit in specialized care (cost per visit)	2018	273 kr	27 €	Swedish Association of Local Authorities and Regions. The max co-payment amount for outpatient care was set to 1100 SEK per year. Only one region in Sweden has a max co-payment less than 1100 SEK; so it was assumed 1100 SEK for the entire country. Swedish Association of Local Authorities and Regions^21^
Cost per month for natalizumab	2018	15,839 kr	1544 €	Treatment with natalizumab is every 4th week (i.e. one per month);^23^ therefore, the cost per month of natalizumab was assumed to be the price for one dose of natalizumab (pharmacy's retail price),^23^ excluding the cost of administration. The latter is partially captured in this study as an outpatient visit to the hospital, excluding infusion visits with a nurse, which are unfortunately not in the NPR.
Cost per month for rituximab	2018	2022 kr	197 €	Rituximab is used off-label in the treatment of MS; therefore, the exact treatment dosing was not available in the Swedish guidelines for MS treatment. A recent published study in Sweden regarding the use of rituximab for PwMS indicated that the drug dose is 500mg to 1000mg per treatment regime (here we assumed the mean, i.e 750mg per treatment regime), and the mean treatment interval for RRMS PwMS was 7.2 months per year.^24^ The cost per month was then assumed to be 1.5 times the pharmacy's retail price per 500mg of rituximab injection, which was taken from FASS (9703.61 SEK),^25^ divided with the frequency of treatment in months (frequency: every 7.2 months).^24^
Monthly salary including employer contributions	2006	40,930 kr	4248 €	The average age-adjusted monthly wage (2018 values) for all employment types was retrieved.^26^ It was multiplied with the annual employer contributions, available from the Swedish Tax Authority.^27^ The final salary was then inflated to 2018 prices using annual Harmonized Indices of Consumer Prices [HICP] for healthcare available from Eurostat.^28^
	2007	41,049 kr	4260 €	
	2008	40,042 kr	4156 €	
	2009	44,583 kr	4627 €	
	2010	44,080 kr	4575 €	
	2011	44,730 kr	4642 €	
	2012	44,748 kr	4644 €	
	2013	45,051 kr	4676 €	
	2014	46,120 kr	4787 €	
	2015	50,300 kr	5221 €	
	2016	48,146 kr	4997 €	

^a^ The annual exchange rate for 2018 from SEK to Euros that was used was 10.2583. Source: Eurostat, Annual Exchange Rates Euro/ECU.

Inpatient and outpatient costs were calculated by multiplying the inpatient stays and outpatient visits during a calendar year, available in the NPR, with their observed nationwide weight for each diagnosis-related group (DRG) and the national cost per 1.0 DRG point.^5^ Co-payment costs were calculated from the use of inpatient stays and outpatient visits as the sum of patient fees for inpatient and outpatient healthcare during each calendar year. The reimbursement period for patient fees was assumed to start on 1 January, and co-payments were set to zero after the accumulated fees had reached the co-payment ceiling each year (see Table 1).

The cost of prescribed dispensed drugs was derived from the SPDR, for each calendar year. These costs included both the cost reimbursed by the county and the co-payment paid by the patient. Then the annual cost of drugs was calculated by summing all costs for dispensed drugs per individual occurring during each calendar year.

The cost of DMTs not available in the SPDR (natalizumab and rituximab for the study period), was calculated using information from the SMSreg (all PwMS receiving natalizumab in Sweden are followed in the SMSreg;^29^ rituximab use was also based on information from the SMSreg for patients included in the register). The number of months on these treatments, calculated taking the end minus the start date for treatment, was multiplied with the cost per month of treatment (Table 1).

The total per patient per year healthcare costs were calculated by adding the costs of inpatient and outpatient healthcare, co-payments, and drugs (including both DMTs and non-DMTs).

Productivity losses were measured based on SA and DP information, using the human capital approach.^30^ All people living in Sweden (≥16 years old) with income from work or unemployment benefits can be granted SA if their work capacity is reduced due to disease or injury. The first day of a SA spell is a waiting day (100% loss of income). Income loss is reimbursed by the employer during days 2 to 14 and after that, by the Swedish Social Insurance Agency. Therefore, the Swedish Social Insurance Agency has no information on SA spells shorter than 15 days for individuals with income from work. For individuals on unemployment benefits, the Swedish Social Insurance Agency pays the benefits from day two.^31^ In order to prevent bias in relation to employment status in the calculation of SA days, only SA spells >14 days were included.

Regarding DP, all people aged 19–65 years can be granted DP if their work capacity is long-term or permanently reduced.^31^ Both SA and DP can be granted for full-time (100%) or part-time (75%, 50%, or 25%) of ordinary work hours.^31^ Therefore, it is possible to have both partial SA and DP simultaneously. In order to handle this and not overestimate the productivity losses, we calculated the net days of SA and DP by using the percentage of the gross days, e.g., 2 absence days at 50% were combined to 1 net day.

To calculate productivity losses, the sum of SA and DP net days per year were used, multiplied with the age-adjusted mean salary, adding the social security contributions made by employers (Table 1).

All costs were inflated to 2018 prices, and were converted to Euros (EUR) (Table 1).

### Analyses

Descriptive statistics were calculated regarding sociodemographic characteristics, multi-morbidity, disability progression, and average COI per patient and year for each of the two treatment groups.

Pearson's chi-squared test^32^ and the likelihood ratio test^32^ were used to explore whether the observed sociodemographic and multi-morbidity differences across the two groups were statistically significant (p-value<0.05).

Two-tailed Student's T-tests, with unequal variance^33^ were used to assess the significance (p-value<0.05) of the disability and cost differences across the two groups.

The mean cost per patient per year, and per group, with 95% confidence intervals (95% CIs), were calculated using a single-distribution generalized linear model with a log link^34^ and with the assumption that costs follow the Poisson distribution. The choice of the distribution reflected the zero costs several individuals had in different cost categories, and that data used in this study (skewed, count or cost data that will always be ≥0), and the need to have conservative cost estimates, avoiding potential overestimation.^35,36^ The annual cost trends were adjusted for the progression of disability over time using the average annual EDSS score per group as the disability measure.

## Results

**Figure 1.**
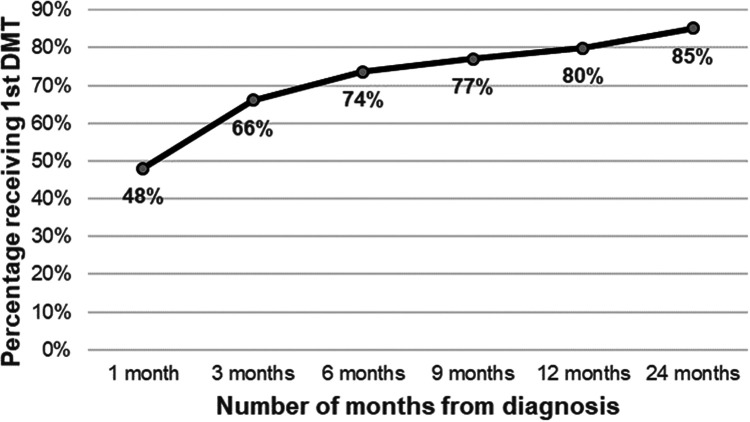
Cumulative percentage of the study cohort receiving their 1st DMT, by months since diagnosis.

In total, 1562 individuals with RRMS receiving their first DMT in 2006–2009 were included. Of them, 74% (n = 1150) received their first DMT within 6 months of the MS diagnosis (Figure 1), while the remaining (n = 412) initiated treatment after this time point. Sex, country of birth, and family situation differed significantly across the two groups (Table 2). In the early treatment group more were women, Swedish born (vs. other nationalities), married/cohabitating with children at home and single without children (vs. married/cohabitating without children or single with children, or young individuals below the age of 21 years living at home). The baseline EDSS score was similar for both groups; higher EDSS score progression was observed for the late treatment group (Figure 1 in the supplementary material).

**Table 2.** Sociodemographic and multi-morbidity characteristics at year of treatment start (index year), by early vs. late treatment groups.

	Early Treatment Cohort	Late Treatment Cohort	Pearson's Chi-Square (p-value)	Log-likelihood test Chi-Square (p-value)
	N = 1150	N = 412		
	n(%)^a^	n(%)^a^		
**Sex**			4.57 (0.033)	4.68 (0.03)
Women	840 (73.04)	323 (78.4)		
Men	310 (26.96)	89 (21.6)		
**Age at year of treatment start (index year)**			1.76 (0.624)	1.77 (0.621)
20-29	307 (26.7)	119 (28.88)		
30-39	437 (38)	162 (39.32)		
40-49	314 (27.3)	101 (24.51)		
50-57	92 (8)	30 (7.28)		
**Education^b^**			3.75 (0.289)	3.68 (0.298)
0-9 years	116 (10.09)	37 (8.98)		
10-12 years	572 (49.74)	192 (46.6)		
>12 years	445 (38.7)	177 (42.96)		
**Country of birth^b^**			9.43 (0.0241)	9.64 (0.022)
Sweden	1020 (88.7)	356 (86.41)		
Nordic countries (except Sweden)	20 (1.74)	13 (3.16)		
EU27 (except Denmark, Finland, and Sweden)	29 (2.52)	4 (0.97)		
Rest of the world	64 (5.57)	33 (8.01)		
**Type of living area^b^**			1.07 (0.586)	1.08 (0.584)
Big cities	452 (39.89)	164 (40.39)		
Medium sized cities	388 (34.25)	147 (36.21)		
Rural areas	293 (25.86)	95 (23.4)		
**Family situation^b, c, d^**			23.11 (0.0001)	25.09 (<.0001)
Married or cohabitant, no children <18 at home	90 (7.94)	54 (13.3)		
Married or cohabitant; children <18 at home	445 (39.28)	176 (43.35)		
Single, no without children <18 at home	478 (42.19)	142 (34.98)		
Single, children <18 at home	79 (6.97)	31 (7.64)		
Youth (aged 18-20 years) living at home	41 (3.62)	<8 (0.74)		
**Any commorbidities**			0.08 (0.779)	0.08 (0.777)
Yes	1125 (97.83)	404 (98.06)		
No	25 (2.17)	8 (1.94)		
**Anxiety/Depression**			2.10 (0.147)	2.23 (0.135)
Yes	66 (5.74)	16 (3.88)		
No	1084 (94.26)	396 (96.12)		

^a^ The percentages are calculated as n divided with the total n in each cohort if not otherwise indicated.

^b^ The total number of individuals with this type of information were n = 1133 for the early treatment group, and n = 406 for the late treatment group; 23 individuals in total had missing information.

^c^ Only cohabitants with children in common are registered as cohabitants. Otherwise they are registered as single.

**Figure 2.**
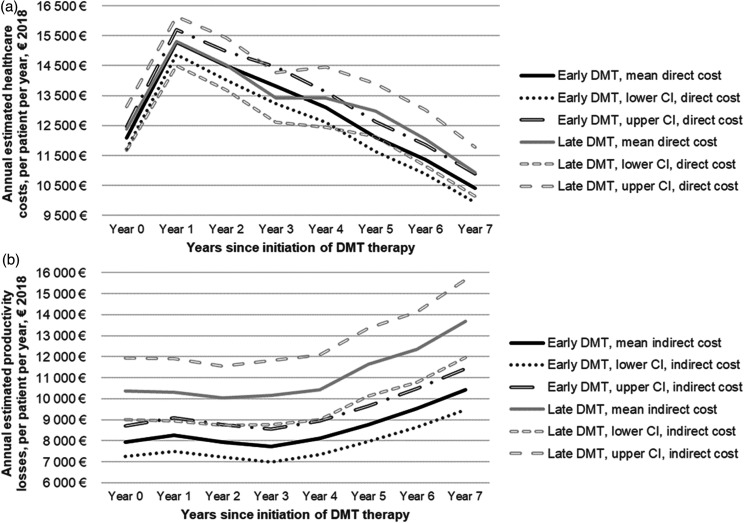
(a, b) COI progression (estimated mean from the regressions) from baseline (index year) to the end of follow-up, by early vs. late treatment groups, adjusted for disability progression (mean annual EDSS score for each group) during the follow-up A) Healthcare costs. B) Productivity losses.

In Table 3 and in Figures 2a-b in the supplementary material, the unadjusted mean per patient per year COI for MS is presented over the 8-year follow-up period for both treatment groups. Figures 2a-b show the disability adjusted healthcare costs and productivity losses over time. Disability adjusted costs were also computed for all the cost components (Figures 3a-f in the supplementary material).

**Table 3.** Mean costs per patient per year, mean costs [95% confidence intervals] in € 2018 prices (crude observed means and confidence intervals, unadjusted), by early vs. late treatment groups.

		**Year 0^a^**	**Year 1**	**Year 2**	**Year 3**	**Year 4**	**Year 5**	**Year 6**	**Year 7**
**Early treatment group (≤ 6 months from diagnosis; N = 1150)**									
	Inpatient costs	2110 [1871-2350]	1206 [947-1464]	1460 [1169-1752]	1443 [979-1907]	1449 [1168-1731]	1222 [965-1479]	1150 [906-1393]	1045 [843-1248]
	Outpatient costs	1513 [1429-1598]	1061 [994-1128]	1169 [1086-1251]	1212 [1136-1289]	1420 [1319-1521]	1450 [1357-1543]	1561 [1462-1660]	1659 [1535-1783]
	Co-payments	110 [107-113]	70 [67-73]	67 [64-69]	64 [61-67]	68 [65-71]	67 [65-70]	68 [65-71]	67 [64-70]
	Drug costs	8387 [8111-8663]	12,986 [12,696-13,276]	11,827 [11,485-12,170]	11,130 [10,756-11,503]	10,202 [9804-10,600]	9412 [8990-9833]	8622 [8195-9050]	7659 [7234-8085]
	**Total healthcare costs**	**12,121 [11,731-12,512]**	**15,323 [14,900-15,745]**	**14,523 [14,036-15,010]**	**13,849 [13,225-14,474]**	**13,139 [12,626-13,652]**	**12,151 [11,645-12,657]**	**11,401 [10,908-11,894]**	**10,430 [9959-10,902]**
	SA costs	8379 [7632-9126]	7844 [7030-8659]	6110 [5397-6823]	4643 [4006-5280]	4302 [3679-4926]	4297 [3687-4907]	4068 [3469-4667]	4129 [3511-4747]
	DP costs	278 [92-464]	1064 [716-1412]	2349 [1848-2850]	3623 [3007-4239]	4535 [3848-5222]	5254 [4514-5993]	6359 [5532-7186]	7299 [6395-8203]
	**Total productivity losses**	**8657 [7896-9418]**	**8908 [8030-9787]**	**8459 [7609-9308]**	**8266 [7399-9133]**	**8837 [7944-9731]**	**9551 [8618-10484]**	**10,427 [9419-11,436]**	**11,428 [10,369-12,488]**
**Late treatment group (> 6 months from diagnosis; N = 412)**									
	Inpatient costs	1603 [1142-2065]	1326 [834-1818]	1674 [1169-2179]	1268 [874-1662]	1525 [800-2249]	1580 [1058-2102]	1342 [856-1827]	1045 [680-1410]
	Outpatient costs	1011 [919-1104]	906 [809-1004]	1106 [963-1248]	1213 [1075-1352]	1268 [1128-1407]	1502 [1341-1664]	1555 [1373-1737]	1632 [1469-1795]
	Co-payments	82 [77-87]	64 [59-68]	66 [61-71]	64 [60-69]	66 [61-71]	72 [67-77]	69 [64-73]	68 [64-73]
	Drug costs	9711 [9186-10236]	13,036 [12,465-13,607]	11,699 [11,049-12,350]	10,882 [10,187-11,578]	10,588 [9882-11,293]	9866 [9149-10,583]	9124 [8386-9861]	8214 [7471-8958]
	**Total healthcare costs**	**12,407 [11,669-13,145]**	**15,332 [14,509-16,155]**	**14,545 [13,661-15,430]**	**13,428 [12,598-14,258]**	**13,446 [12,434-14,458]**	**13,021 [12,114-13,928]**	**12,089 [11,143-13,035]**	**10,960 [10,126-11,793]**
	SA costs	6477 [5264-7690]	5418 [4276-6560]	3906 [3004-4808]	3259 [2353-4165]	2991 [2227-3756]	4000 [3064-4937]	4253 [3250-5257]	4800 [3692-5908]
	DP costs	4700 [3524-5876]	5566 [4270-6863]	6738 [5335-8142]	7456 [5982-8929]	8241 [6675-9806]	8729 [7130-10329]	9303 [7637-10968]	10,081 [8319-11,842]
	**Total productivity losses**	**11,177 [9572-12,782]**	**10,985 [9329-12,641]**	**10,644 [9048-12,241]**	**10,714 [9056-12,372]**	**11,232 [9546-12,919]**	**12,730 [10,953-14,507]**	**13,556 [11,707-15,405]**	**14,880 [12,930-16,831]**

^a^ This is the year of initiation of the DMT therapy (index year).

For both groups, medications were the main cost driver for healthcare costs while DP was the driver for productivity losses. SA costs decreased over time while DP costs increased, indicating a shift from short-term to long-term productivity losses. Both treatment groups had similar healthcare costs (p-value>0.05), which increased the first year after diagnosis, and then decreased for the rest of the follow-up period. Those receiving treatment late had statistically significant higher productivity losses throughout the study period (p-value = 0.001).

## Discussion

In this register-based prospective cohort study, we explored the development of the MS COI among newly diagnosed PwMS in Sweden over time in relation to how long after diagnosis PwMS received their first DMT, adjusting for MS disability progression.

Those receiving the first DMT within 6 months after diagnosis had lower productivity losses over time, possibly through maintained work capacity. Even after adjusting for differences in MS disability between the two groups, fewer PwMS in the early treatment group were on DP over time. While having DP already during the time when receiving the first DMT (at Year 0 in our study) could be related to MS symptom onset prior to the actual diagnosis of the disease,^37,38^ the timing of DMT initiation can still explain part of this difference. Some PwMS in the late treatment group have received their first DMT several years after diagnosis; while 85% of our total study cohort have received therapy by 24 months after diagnosis (Figure 1); 15% of individuals received it sometime afterwards. Therefore, the occurrence of DP during Year 0 for this group could potentially be linked to the presence of important MS symptoms and/or progression. In addition, intervening with a DMT as early as possible can result in fewer MS relapses, and/or postpone MS progression. The presence of MS symptoms and the consequences of MS progression, such as fatigue, weakness, and cognitive and motor impairment, have been stated as preventing remaining in paid work.^4,39^

Both groups had similar healthcare costs over time, indicating that it is more likely that MS symptoms requiring specialized medical attention and other comorbidities occur independent of the time of treatment initiation. The cost of drugs (referring to any drugs MS patients have received, not only DMTs) was slightly higher among those initiating treatment late (crude cost estimates; p-value>0.05) during the first year of follow-up, and similar in both groups thereafter.

The cost of drugs in this study was the main driver of healthcare costs in both groups. As previous studies have shown,^8,9^ new DMTs have changed the treatment landscape of MS. Accordingly, in the last two decades drugs have become main cost drivers in MS, while the need for expensive inpatient healthcare has declined.

The overall trends of healthcare costs and productivity losses observed here are in line with previous COI studies in MS with similar longitudinal designs.^5–7^ In addition, in line with our findings, previous studies suggest that initiating treatment as early as possible is associated with better clinical outcomes and ability to maintain employment^40^ for PwMS over time.^11,12^ While these studies focused on treatment early from MS onset and rather than soon after diagnosis, i.e. having a different study design than the present study, they nevertheless point to the same conclusion, that early treatment is beneficial to patients by slowing MS progression.^11,12^

Moreover, our study aimed to present the benefit of early treatment in the overall COI of MS, which is something that has been studied sparsely so far, and not involving longitudinal COI data with multiple years of follow-up. We found no other longitudinal study which uses observed data, measuring the COI progression in relation to time of treatment initiation after MS diagnosis. One US study has shown that receiving DMTs before MS diagnosis vs. afterwards, can have a positive impact on the COI of MS.^41^ However, they only followed the patients for one year after treatment initiation,^41^ and they did not take into consideration any differences in the baseline MS disability of PwMS in the groups, nor its progression. In addition, one study used a health economic model, to estimate costs and effects from MS, concluding that early treatment with DMTs was cost-effective.^42^

### Strengths and limitations

Main strengths of this study are the prospective cohort design, with eight years of follow-up, and that data from administrative registers could be used, instead of self-reported information. All people with RRMS in the SMSreg receiving their first DMT in 2006–2009 could be followed for eight years in nationwide registers, eliminating both drop outs and recall biases that some previous MS studies had due to using self-reported information.^7^ While this is an important strength, unlike those previous studies, registers did not contain information regarding primary healthcare, rehabilitation measures, home help, and home investments to improve mobility, to include in our COI calculations. Such information could complement our results, showing how MS symptoms and multi-morbidity can impact the overall healthcare costs in MS, based on the treatment timing as well. In addition, no information on multi-morbidity reported by patients was available for our study; our multi-morbidity definition was based on the Rx-Risk Comorbidity Index,^19^ for which drug use data were used to measure morbidity, which can provide limited information for multi-morbidity.

While eight years of follow-up can give an indication of whether early or late therapy can have an impact on the COI in MS over time, it would be useful to have a longer follow-up to identify any future COI differences. Treatment switches and discontinuation can also play a role alongside the timing of treatment initiation in the overall COI progression in MS. However, such information was not captured in the findings of this study.

High quality and robust information from the MiDAS register in Sweden was available, capturing with accuracy the number of SA and DP net days per year. This allowed for detailed calculations of the productivity losses, which are considered the main long-term driver of the COI in MS.^7^ However, a limitation of this study is that we did not have information on short-term SA-spells (≤14 days). Therefore, these shorter absences which were not quantified into productivity losses, possibly leading to the underestimation of these costs. In addition, these shorter SA spells are mainly not related to MS, it could thus be assumed not to differ substantially between the two studied groups.

Moreover, any reductions in productivity while being present at work that could potentially be related to the presence of MS were not quantified. Furthermore, productivity losses incurred by partners of PwMS, i.e., informal care costs, were not included in the COI calculations. While measuring such costs was beyond the aim of this study, they are an important cost component when defining the overall COI of MS.^7^

Similar to what previous COI of MS studies have done,^7^ we used the EDSS score to explore disability progression for PwMS in the two treatment groups linking it to the COI. However, EDSS captures only limited domains of disability among PwMS, e.g., fatigue is missing, and only at the point in time of the patient's disability assessment. The use of additional clinical measures could complement our analysis regarding the progression of MS disability, allowing a better correlation of disability with the timing of treatment initiation, and eventually the COI.

To allow for the difference of the timing of treatment initiation in line with our research objective, the healthcare cost outcomes and productivity losses were adjusted for by EDSS, for every year of follow-up. This method has the limitation of minimizing the potential health-improving effects of the DMTs in the resultant costs. Therefore, the unadjusted healthcare costs and productivity losses were also provided in the supplement, to demonstrate the treatment effects over time in the cost progression.

While nationwide registers with robust data were used in this study,^15^ the generalizability of our findings can be limited. The study cohort was taken from SMSreg, which at that time (2006–2009) had approximately 50%-60% coverage of all PwMS in Sweden.^43^ With caution and assuming no distinct differences in the sociodemographic characteristics, multi-morbidity, and disability with the PwMS that our study did not include, our findings could be generalizable to all PwMS in Sweden. Generalization and applicability of our findings to other countries may not be possible, considering the differences in the organization of healthcare and social security systems.

## Conclusions

In this study, register data with nationwide coverage were used to explore the association between the timing of the first DMT initiation among PwMS with the COI. Productivity losses for PwMS who started their therapy early were significantly lower. Therefore, suggesting that by intervening as early as possible to stop MS progression, there is a lower impact on the work capacity of PwMS in terms of lower SA and DP days. COI serves as an objective measure of the burden of MS, reflecting how morbidity and disability linked to MS evolve throughout patients’ days and over many years, as well as highlighting the advantage of early vs. late DMT initiation in MS.

## Supplemental Material

sj-docx-1-mso-10.1177_20552173221092411 - Supplemental material for Early vs. late treatment initiation in multiple sclerosis and its impact on cost of illness: A register-based prospective cohort study in Sweden Click here for additional data file. Supplemental material, sj-docx-1-mso-10.1177_20552173221092411 for Early vs. late treatment initiation in multiple sclerosis and its impact on cost of illness: A register-based prospective cohort study in Sweden by Korinna Karampampa, Hanna Gyllensten, Chantelle Murley, Kristina Alexanderson, Andrius Kavaliunas, Tomas Olsson, Ali Manouchehrinia, Jan Hillert and Emilie Friberg in Multiple Sclerosis Journal – Experimental, Translational and Clinical
