# Supplementary material for: Early vs. late treatment initiation in multiple sclerosis and its impact on cost of illness: A register-based prospective cohort study in Sweden
Source: Mult Scler J Exp Transl Clin. 2022 Apr 24;8(2):20552173221092411. doi: 10.1177/20552173221092411 (PMC9044795; doi:10.1177/20552173221092411)
Supplement: sj-docx-1-mso-10.1177_20552173221092411 - Supplemental material for Early vs. late treatment initiation in multiple sclerosis and its impact on cost of illness: A register-based prospective cohort study in Sweden [file sj-docx-1-mso-10.1177_20552173221092411.docx]

# Supplementary online material

Supplementary Figure 1: EDSS score progression from baseline (index year) to the end of follow-up, by early vs. late treatment groups


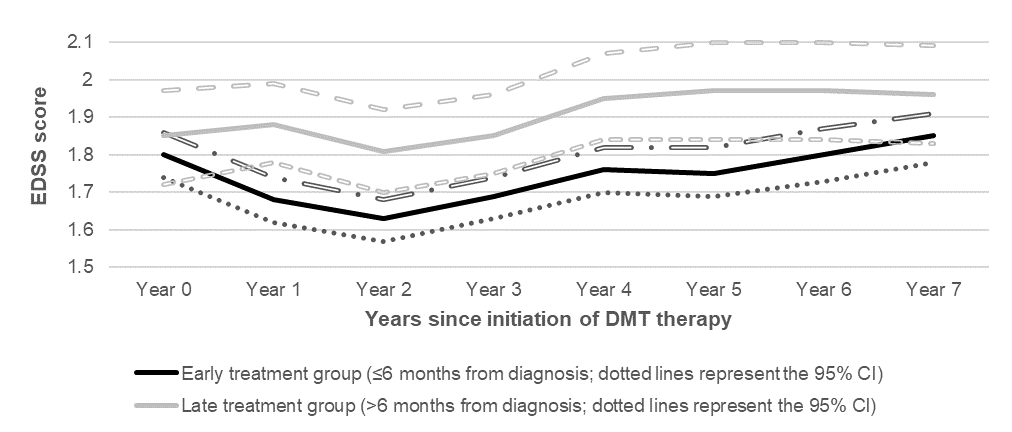


Supplementary Figure 2 (a, b): COI progression, unadjusted means, from baseline (index year) to the end of follow-up, by early vs. late treatment groups

1. **Healthcare costs**


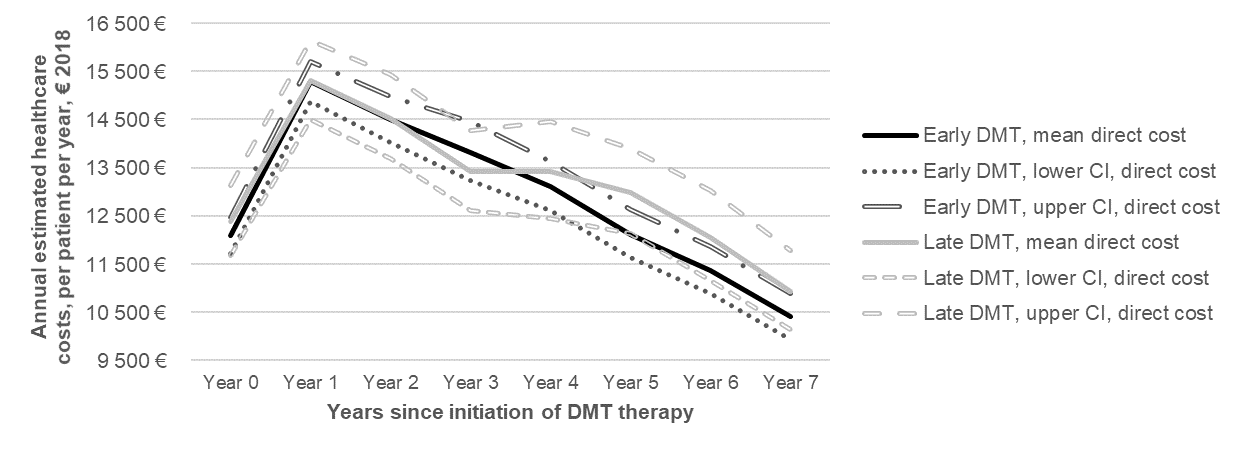


1. **Productivity losses**

**
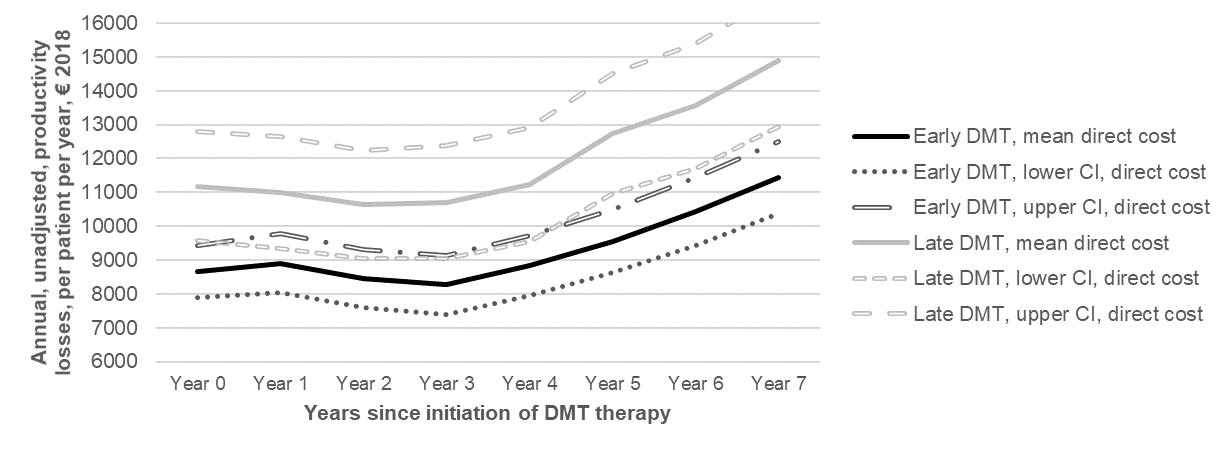
**

Supplementary Figure 3 (a-f): Progression (estimated mean from the regressions) for all cost components, from baseline (index year) to the end of follow-up, by early vs. late treatment groups, adjusted for disability progression (mean annual EDSS score for each group) during the follow-up

1. **Inpatient costs**

**
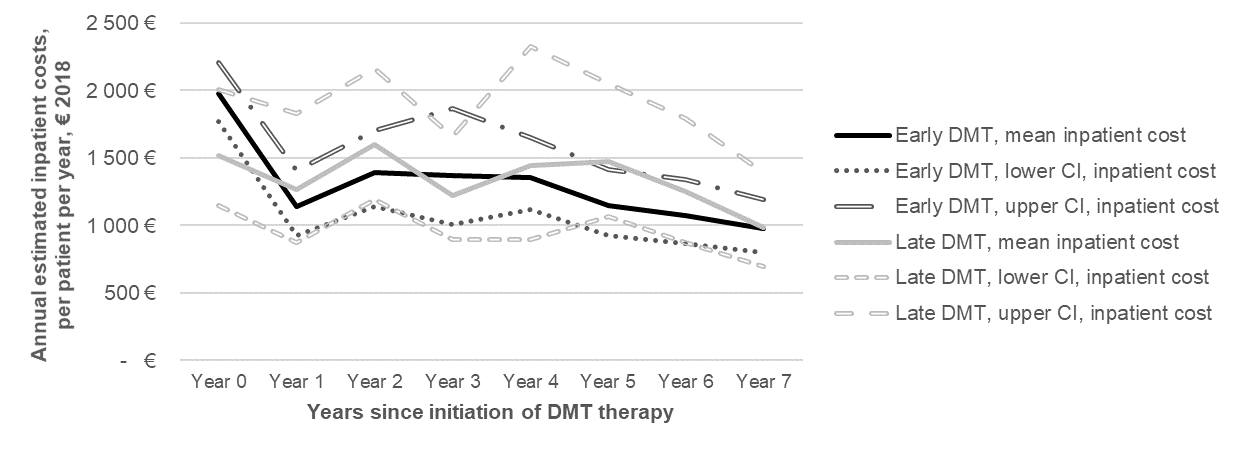
**

1. **Outpatient costs**

**
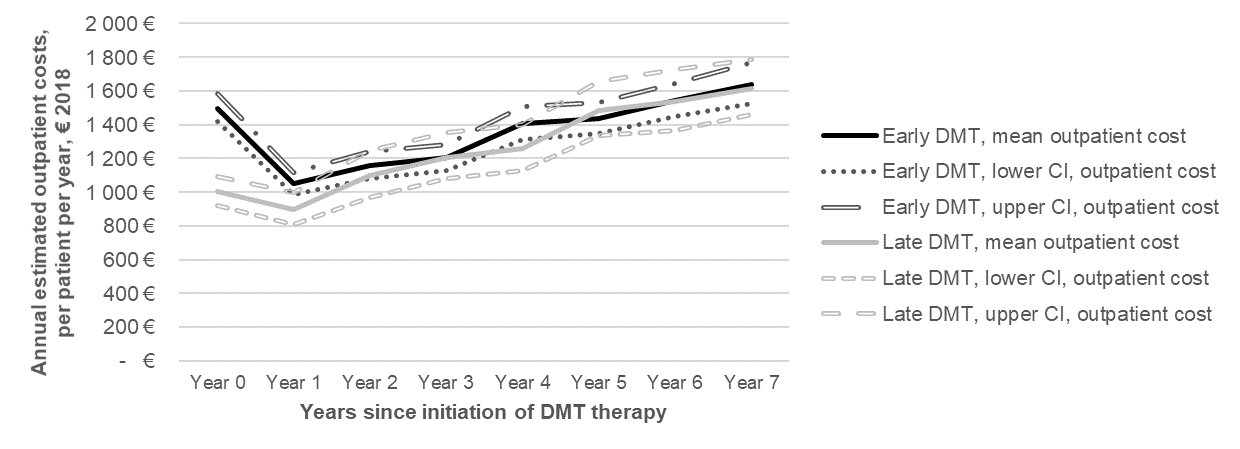
**

1. **Co-payments**

**
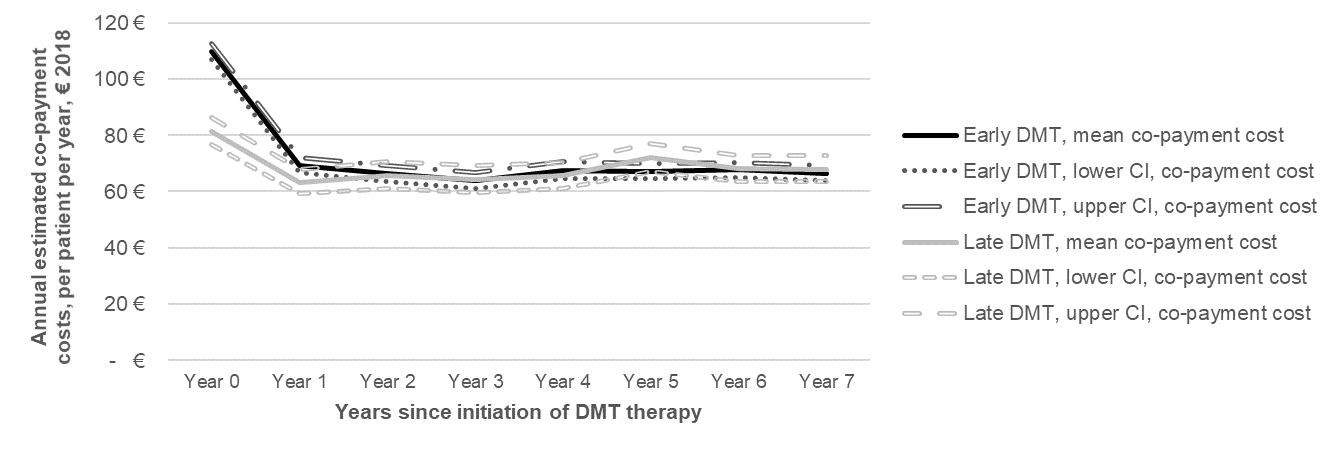
**

1. **Drug costs**

**
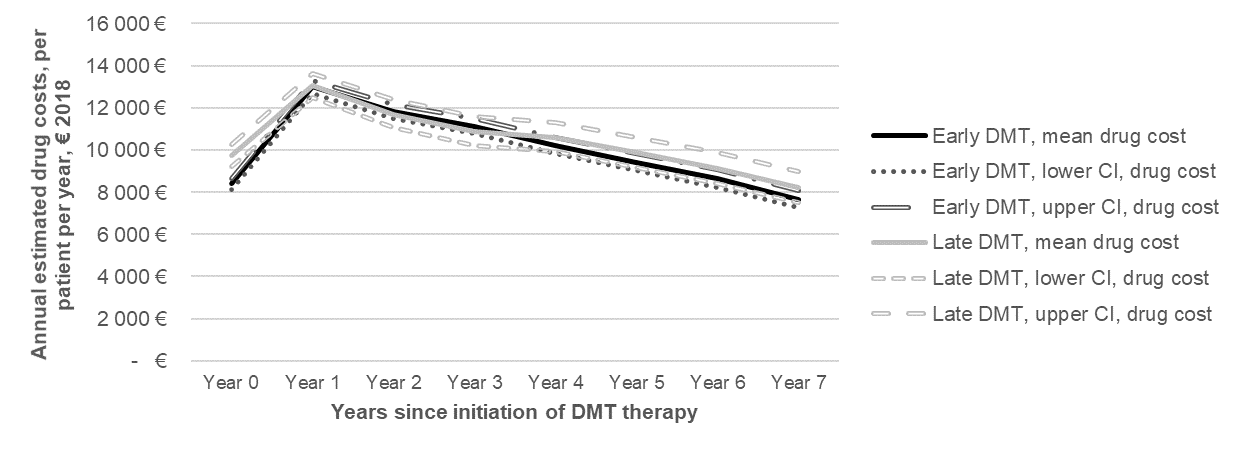
**

1. **Sickness absence costs**

**
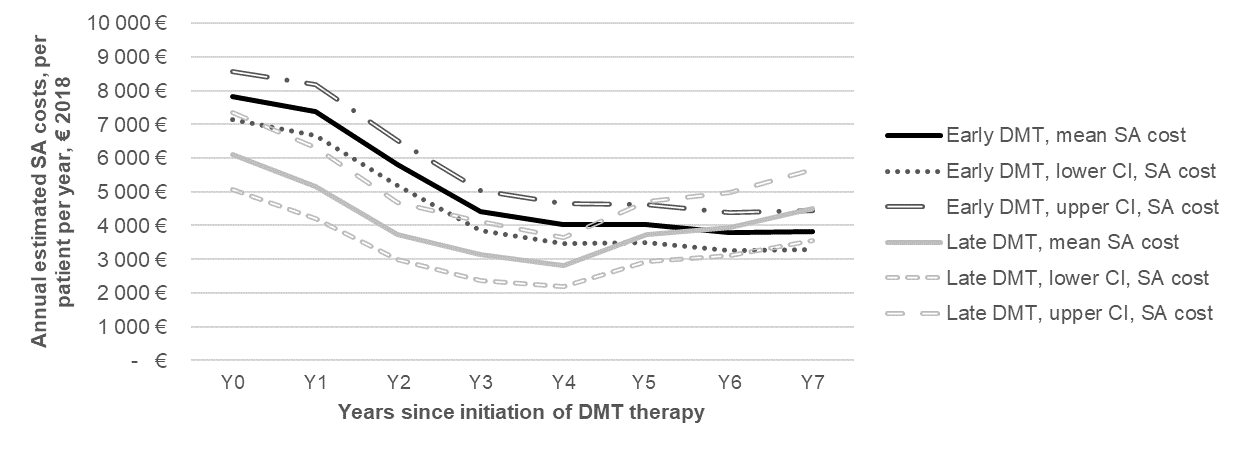
**

1. **Disability pension costs**

**
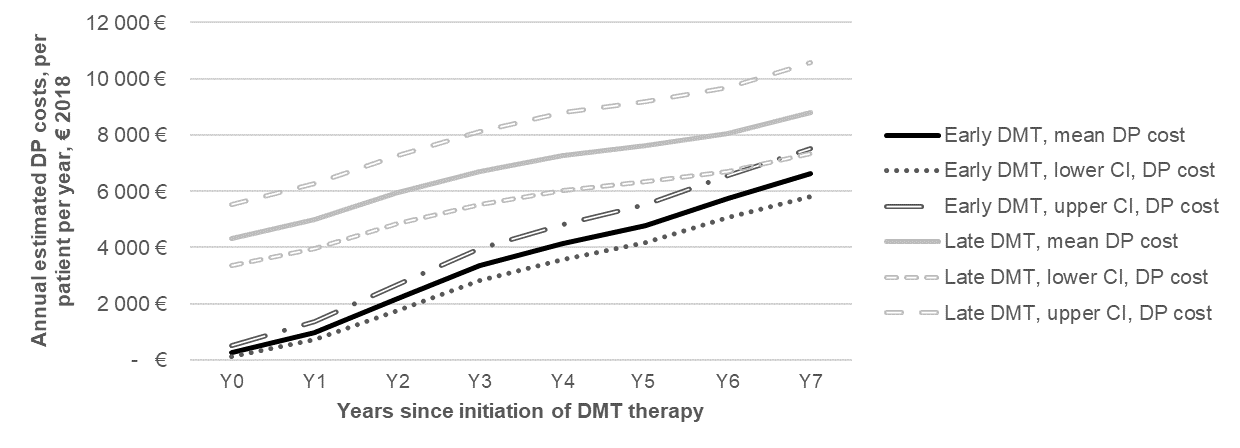
**
